# By reducing global mRNA translation in several ways, 2-deoxyglucose lowers MCL-1 protein and sensitizes hemopoietic tumor cells to BH3 mimetic ABT737

**DOI:** 10.1038/s41418-018-0244-y

**Published:** 2018-12-11

**Authors:** Maximilien Tailler, Lisa M. Lindqvist, Leonie Gibson, Jerry M. Adams

**Affiliations:** 1 The Walter and Eliza Hall Institute of Medical Research, 1G Royal Parade, Parkville, VIC 3052 Australia; 2 Department of Medical Biology, The University of Melbourne, Parkville, Melbourne, VIC 3010 Australia; 3 Present Address: CSL Ltd, Parkville, VIC Australia

**Keywords:** Gene regulation, Cancer metabolism

## Abstract

Drugs targeting various pro-survival BCL-2 family members (‘‘BH3 mimetics’’) have efficacy in hemopoietic malignancies, but the non-targeted pro-survival family members can promote resistance. Pertinently, the sensitivity of some tumor cell lines to BH3 mimetic ABT737, which targets BCL-2, BCL-XL, and BCL-W but not MCL-1, is enhanced by 2-deoxyglucose (2DG). We found that 2DG augmented apoptosis induced by ABT737 in 3 of 8 human hemopoietic tumor cell lines, most strongly in pre-B acute lymphocytic leukemia cell line NALM-6, the focus of our mechanistic studies. Although 2DG can lower MCL-1 translation, how it does so is incompletely understood, in part because 2DG inhibits both glycolysis and protein glycosylation in the endoplasmic reticulum (ER). Its glycolysis inhibition lowered ATP and, through the AMPK/mTORC1 pathway, markedly reduced global protein synthesis, as did an ER integrated stress response. A dual reporter assay revealed that 2DG impeded not only cap-dependent translation but also elongation or cap-independent translation. MCL-1 protein fell markedly, whereas 12 other BCL-2 family members were unaffected. We ascribe the MCL-1 drop to the global fall in translation, exacerbated for mRNAs with a structured 5′ untranslated region (5′UTR) containing potential regulatory motifs like those in *MCL-1* mRNA and the short half-life of MCL-1 protein. Pertinently, 2DG downregulated two other short-lived oncoproteins, MYC and MDM2. Thus, our results support MCL-1 as a critical 2DG target, but also reveal multiple effects on global translation that may well also affect its promotion of apoptosis.

## Introduction

Perturbed regulation of apoptosis can promote cancer and affect its treatment [1, 2]. The interactions of three BCL-2 protein sub-families govern its regulation. Once activated, the critical effectors BAX and BAK form oligomers that permeabilize the mitochondrial outer membrane, triggering caspase-mediated cellular demolition. Pro-survival members, such as BCL-2, BCL-XL, and MCL-1, prevent BAX and BAK activation until overwhelmed by their apoptosis-signaling BH3-only relatives [1, 2].

An exciting new approach to cancer therapy, particularly promising for hematopoietic malignancies, is targeting one or more pro-survival BCL-2 family member with small molecules that engage pro-survival proteins similarly to their natural BH3-only antagonists [1, 3]. The first potent authentic ‘‘BH3 mimetic’’, ABT737 [4], inhibits BCL-2, BCL-XL, and BCL-W but not MCL-1 [4, 5]. Underscoring the potential of BH3 mimetics, in 2016 the FDA approved the BCL-2-specific ABT199 (venetoclax) for treating refractory chronic lymphocytic leukemia. However, in tumor cells treated with ABT737, MCL-1 is a prime resistance factor [5–8], emphasizing the need for agents providing complementary function.

Multiple studies report that 2-deoxyglucose (2DG) aids ABT737 to induce apoptosis in certain cell lines from solid tumors [9] or hemopoietic malignancies [10–12]. Moreover, earlier seminal studies in a mouse lymphomagenesis model had linked initiation of protein translation, the mechanistic target of rapamycin complex 1 (mTORC1) and the MCL-1 level [13, 14].

Because 2DG is relatively non-toxic, its combination with a BH3 mimetic might find clinical utility [15, 16], but how 2DG contributes to tumor cell death is incompletely understood. As well as impeding glycolysis, 2DG impedes protein glycosylation in the endoplasmic reticulum (ER), perhaps by inducing an unfolded protein response (UPR) [17, 18], and multiple factors influence the dominant path, e.g., cell type, oncogenic changes and 2DG concentration [15, 16]. In any case, 2DG lowers MCL-1 protein [9–12, 17, 18], which augments the cytotoxic action of BH3 mimetics like ABT737 [1, 3]. Intriguingly, the lower MCL-1 has been ascribed to attenuated global translation by the AMPK/mTORC1 pathway [19, 20] and/or the ER stress [12, 17, 18].

Here, we explore the basis for the cytotoxic cooperativity of 2DG and ABT737 in several human hematopoietic cell lines. The results support a crucial contribution of reduced MCL-1 to their cooperativity, but also reveal that 2DG impairs global mRNA translation in several ways that likely also affect its cytotoxicity. We propose that features in the *MCL-1* 5′UTR contribute to its reduced translation. Pertinently, the control of translation and its role in cancer development and treatment are under intense study [21–23].

## Results

### 2DG augments apoptosis by BH3 mimetics in certain hemopoietic tumor cell lines

**Fig. 1:**
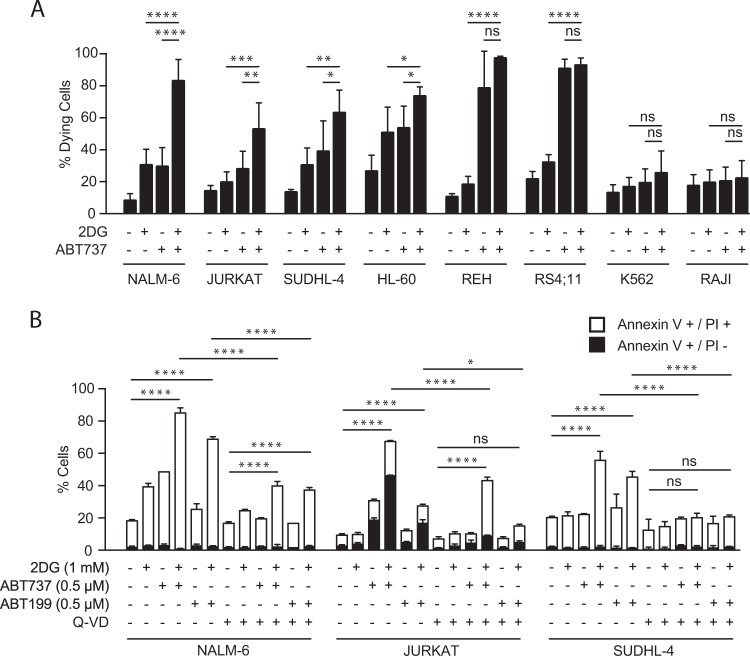
2DG plus a BH3 mimetic promotes cell death in certain hematopoietic tumor cell lines. **a** Flow cytometric analysis of the cell death induced by 2DG (1 mM), ABT7373 (0.5 μM) or their combination in eight hematopoietic tumor cell lines. Cells were treated for 24 h, and cell death analyzed by propidium iodine staining. Data are plotted as mean ± SD. Significance was determined by one-way ANOVA: *P < 0.05, **P < 0.01, ***P < 0.001, ****P < 0.0001; ns, not significant. **b** FACS analysis of cell death protection by caspase inhibitor Q-VD (50 μM) during 24 h treatment with 2DG (1 mM) and BH3 mimetic ABT737 or ABT199 (0.5 μM). Cell death is analyzed by Annexin-V and propidium iodine staining. Data are plotted as mean ± SD. Significance was determined as above

We first determined the efficacy of 2DG plus ABT737 on eight well-characterized human hemopoietic tumor cell lines (Fig. 1a). Their combination notably enhanced killing in pre-B leukemia ALL line NALM-6, the JURKAT T-lymphoma line, and diffuse large B-cell lymphoma line SUDHL-4 but was less effective in the others: ABT737 alone killed effectively in the REH and RS4;11 ALL lines, as did 2DG alone in the pro-myelocytic HL-60 line, whereas the early erythroid K562 and RAJI Burkitt lymphoma lines were refractory even to the combination. Since NALM-6 exhibited the greatest cooperativity, over twice the killing by either single agent (Fig. 1a), we have given it most attention.

The cell death from 2DG + ABT737 was by apoptosis, as co-treatment with the caspase inhibitor Q-VD largely prevented it (Fig. 1b) (see below). Moreover, the cooperativity extended to 2DG plus ABT199, which was nearly as effective, at least with NALM-6 and SUDHL-4 (Fig. 1b). As reported previously [9], the cooperativity developed within 2 or 3 h (Supplementary Fig. S1A), so our mechanistic studies followed 0–6 h of treatment.

### 2DG elicits an integrated stress response involving the ER

With NALM-6, JURKAT and SUDHL-4 cells, 2DG alone killed substantially at higher doses (5–20 mM) (Supplementary Fig. S1B). The apoptosis attributable to impaired glycosylation in the ER, which can induce an unfolded protein response [24], is attenuated by mannose co-treatment [15, 16], as we observed (Supplementary Fig. S1B). We also assessed how mannose or Q-VD affected cytochrome c release in NALM-6 cells treated with the drugs (Supplementary Fig. S1C, D). Since mannose acts upstream of cytochrome c release, whereas caspases act downstream, mannose but not Q-VD prevented its release from mitochondria (Supplementary Fig. S1C). Conversely, both mannose and Q-VD impeded cleavage of Poly(ADP-ribose) polymerase (PARP), a classic apoptosis marker evoked by 2DG + ABT737 but not ABT737 alone (Supplementary Fig. S1D).

**Fig. 2:**
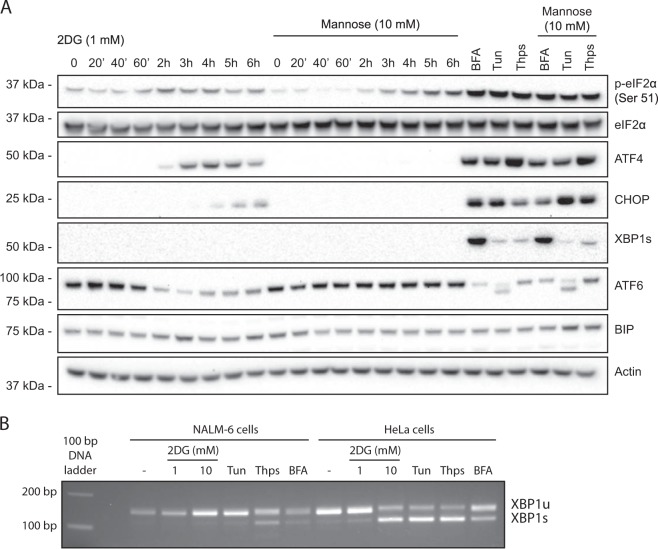
The cell death involves an integrated stress response in the ER. **a** Kinetics of ER stress induction by 2DG, assessed by western blotting in NALM-6 cells treated with 1 mM 2DG for up to 6 h, in the presence or absence of mannose. Positive controls were ER stress inducers brefeldine A (BFA, 2.5μg/mL), tunicamycin (Tun, 1 μg/mL), and thapsigargin (Thps, 1 μM), analyzed at 6 h. Major markers of ER stress evaluated included p-eIF2α (ser51), ATF4, CHOP, the spliced form of XBP1 (XBP1s), ATF6, and BIP (GRP78). Actin served as a loading control. **b** RT-PCR data confirming that NALM-6 cells treated with 1 or 10 mM 2DG for 6 h exhibit unspliced XBP1 mRNA (XBP1u, 149 bp) but not the spliced mRNA (XBP1s, 109 bp). The spliced product is evident after treatment with thapsigargin and (faintly) BFA. HeLa cells yielded XBP1s with 10 mM 2DG and the other stimuli

Since 2DG acts partly through ER stress, we monitored how ER stress markers responded to 2DG over time, or to potent ER stressors for 6 h. Notably, 2DG provoked an integrated stress response (ISR) (Fig. 2a) [25–27]: after 2 to 6 h of 2DG, phosphorylation of eIF2α on serine-51 increased and, concomitantly, the altered translation revealed transcription factor ATF4, and, somewhat later, its downstream target transcription factor C/EBP homologous protein (CHOP). Mannose co-treatment precluded these effects, but not the robust responses evoked by the known stressors, which must induce an ISR by different pathways (Fig. 2a). Notably, 2DG-treated NALM-6 cells did not have a complete ER stress response, e.g., one involving upregulated BIP (GRP78) or spliced XBP-1 protein (XBP-1s) at the times and 2DG concentrations (1 mM or 10 mM) used (Fig. 2a), and reverse transcription (RT)-PCR confirmed that XBP-1 mRNA remained unspliced (Fig. 2b) (see Discussion).

Activation by 2DG of the ISR eIF2α/ATF4/CHOP pathway markedly alters global protein synthesis: to restore homeostasis, it downregulates the predominant cap-dependent translation and upregulates translation of stress-associated transcripts, such as that encoding ATF4 [25–27].

### 2DG reduces MCL-1 protein but leaves 12 BCL-2 relatives unaffected

**Fig. 3:**
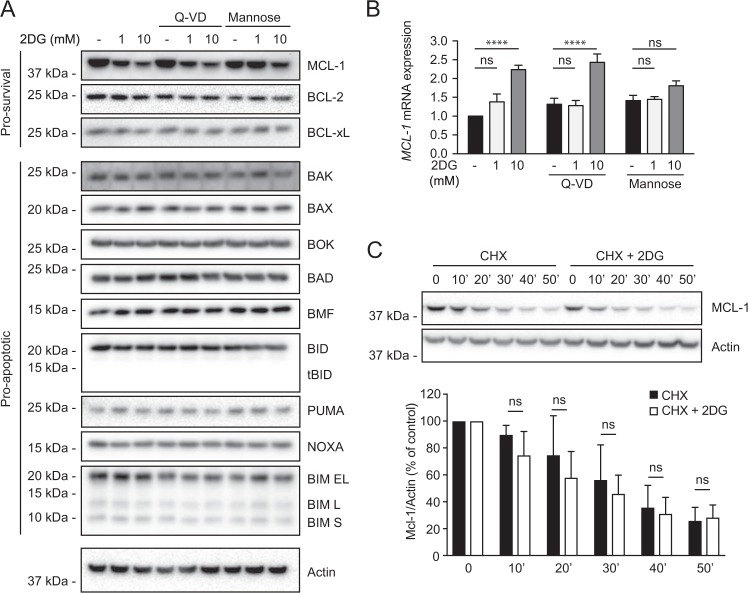
Selective impact of 2DG on the BCL-2 protein family. **a** Abundance of pro-survival and pro-apoptotic proteins of the family, assessed by western blotting, in NALM-6 cells after 6 h of 2DG treatment (1 or 10 mM), showing that only MCL-1 is affected and that mannose (10 mM), but not caspase inhibition (50 μM Q-VD), partially relieves the MCL-1 drop. **b** *MCL-1* mRNA abundance assessed by quantitative RT-PCR in NALM-6 cells treated for 6 h with 2DG (1 or 10 mM) and co-treated as indicated with Q-VD (50 μM) or mannose (10 mM). TRIzol purified mRNA was subjected to random primer RT-PCR, and *MCL-1* mRNA level determined by quantitative PCR, normalized by a geometrical mean of four housekeeping mRNAs (GAPDH, TUBβ1, PPIA, and TBP, see Methods). Data are plotted as mean ± SD. Significance was determined by two-way ANOVA: ****P < 0.0001. **c** 2DG does not accelerate degradation of MCL-1 protein. NALM-6 cells were treated with the protein synthesis inhibitor cycloheximide (CHX, 20μg/mL), in the presence or absence of 2DG (1 mM), and the MCL-1 level assessed every 10 min by western blotting. A representative western blot is shown; the histogram below it quantifies three independent western blots, each normalized to actin. Data are plotted as mean ± SD. Significance was determined by Student’s *t*-test: ns, not significant (*P* > 0.05)

As in various tumor lines [9–12, 17–20, 28], treating NALM-6 for 6 h with 1 or 10 mM 2DG markedly reduced MCL-1 (Fig. 3a, top). It fell by 3 h (Supplementary Fig S2A), whereas pro-survival relatives BCL-2 or BCL-XL were unaffected (Fig. 3a and Supplementary Fig. S2A), and BCL-W was barely detectable even in untreated cells (Supplementary Fig. S2B); lack of an effective antibody precluded analyzing the less well-studied BFL-1. No pro-apoptotic relatives changed (Fig. 3a), including apoptosis effectors BAK or BAX, their close relative BOK, and the six BH3-only proteins analyzed, even though BIM and PUMA can contribute to ER stress responses [29, 30], and NOXA can drive MCL-1 degradation [31] (see Discussion).

### MCL-1 protein drops despite increased *MCL-1* mRNA and unchanged MCL-1 protein degradation

Because MCL-1 expression is regulated at multiple levels [32], we explored different mechanisms for the diminished MCL-1 protein. Quantitative PCR showed that *MCL-1* mRNA did not decrease (Fig. 3b). Indeed, at 10 mM 2DG, its level actually rose, perhaps reflecting a feedback mechanism whereby low MCL-1 protein stimulates *MCL-1* transcription. In any case, the reduced MCL-1 protein is clearly not due to inhibited *MCL-1* transcription, or decreased stability of its mRNA.

Although MCL-1 protein has a short half-life [32], we tested whether 2DG increased its degradation by blocking translation with cycloheximide and monitoring MCL-1 protein after exposure (or not) to 2DG; the ~ 30 min MCL-1 half-life was unaffected by 2DG (Fig. 3c). Accordingly, proteasome inhibitor MG132 simply increased the MCL-1 level in all treatment conditions (Supplementary Fig. S2C).

### 2DG lowers the ATP level and reduces global protein synthesis

**Fig. 4:**
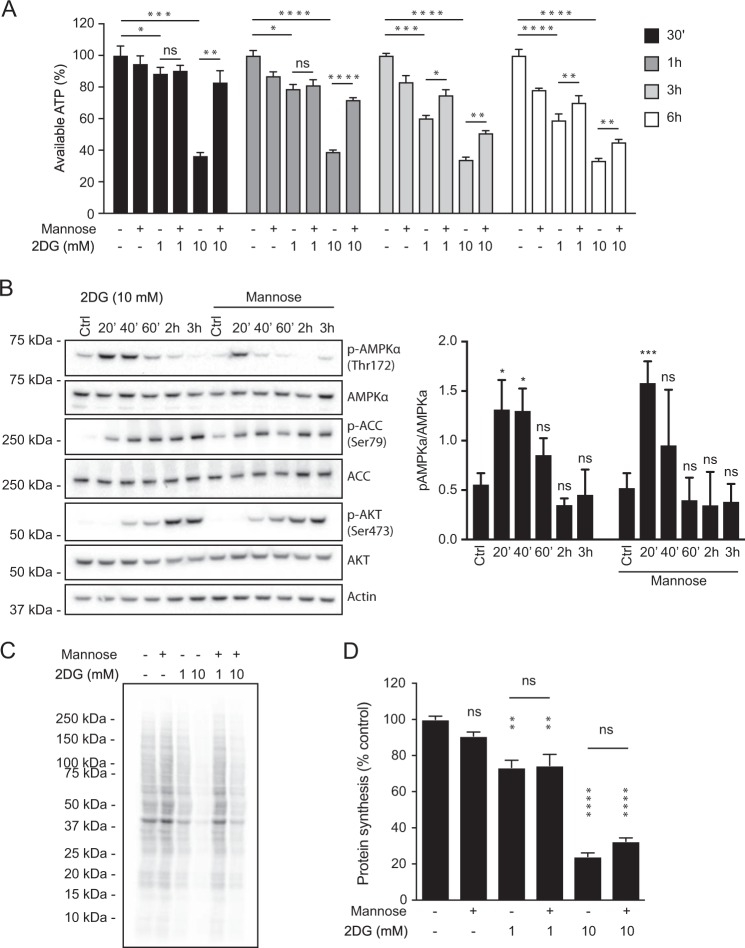
2DG induces a rapid fall in ATP, transient AMPKα activation and reduced global protein synthesis. **a** Quantification of ATP in NALM-6 cells treated for 30 min to 6 h with 1 or 10 mM 2DG, with or without 10 mM mannose. Data are plotted as mean ± SD. Significance determined by one-way ANOVA: *P < 0.05, **P < 0.01, ***P < 0.001, ****P < 0.0001. **b** Immunoblots showing effect of 10 mM 2DG on AMPKα, its substrate ACC and AKT in NALM-6 cells treated 20 min to 6 h, with or without mannose (10 mM) (left panel). Quantification of p-AMPKα in four such immunoblot experiments (right panel) indicates its significant elevation at 20 and 40 min but not thereafter; with mannose, only the rise at 20 min was significant. Data are plotted as mean ± SD. Significance of the difference from the untreated control was determined by one-way ANOVA: *P < 0.05, ***P < 0.001; ns, not significant (*P* > 0.05). **c** 2DG reduces global protein synthesis. NALM-6 cells were treated for 3.5 h with 1 or 10 mM 2DG, with or without mannose (10 mM), and ^35^S-methionine/^35^S-cysteine was then added for 30 min. New protein synthesis, resolved by SDS-polyacrylamide gel electrophoresis, was visualized by subjecting a transfer membrane to a phosphorimager. **d** Quantification of new protein synthesis in three independent experiments like that in **c**, evaluated from the level of ^35^S-methionine/^35^S-cysteine incorporated into polypeptides precipitated by trichloroacetic acid. Data are plotted as mean ± SD. Significance of the difference from the untreated control was determined by one-way ANOVA: **P < 0.01, ****P < 0.0001; ns, not significant (*P* > 0.05)

In keeping with inhibited glycolysis, 1 mM 2DG reduced ATP ~ 12% by 30 min and 40% after 3 and 6 h (Fig. 4a), while 10 mM provoked a precipitous fall of ~ 60% by 30 min and 70% at 3 and 6 h. With 10 mM 2DG, mannose co-treatment markedly attenuated the ATP drop, particularly at 30 min and 1 h (Fig. 4a). The ATP drop is expected to activate energy-sensor AMP-activated Kinase-α (AMPKα) [33]. Indeed, with 10 mM 2DG, its phosphorylation on Thr172 rose significantly by 20 min, and it phosphorylated its classic substrate Acetyl-CoA Carboxylase (ACC) on Ser79 (Fig. 4b). By 60 min, p-AMPKα had returned to the untreated level, but p-ACC remained for hours. With 1 mM 2DG, however, no statistically significant rise in p-AMPKα appeared, due to variability in its untreated level (*n* = 8; data not shown); presumably, 1 mM 2DG could not fully activate AMPKα. Curiously, with 10 mM 2DG, mannose hastened the decline in p-AMPKα (Fig. 4b).

Importantly, 2DG markedly depressed global protein synthesis, as the fainter pattern of newly synthesized S^35^-labeled total protein shows (Fig. 4c), particularly with 10 mM 2DG (compare lanes 1 and 4). Indeed, quantifying acid-precipitable labeled polypeptides (Fig. 4d) revealed that 1 mM 2DG for 4 h reduced protein synthesis by 25% and 10 mM 2DG by 75%. Interestingly, mannose did not significantly blunt this drop. The reduced protein synthesis, at least at 1 mM 2DG, did not affect the cell cycle (Supplementary Fig. S2D).

### 2DG depresses both cap-dependent and elongation or cap-independent protein synthesis

**Fig. 5:**
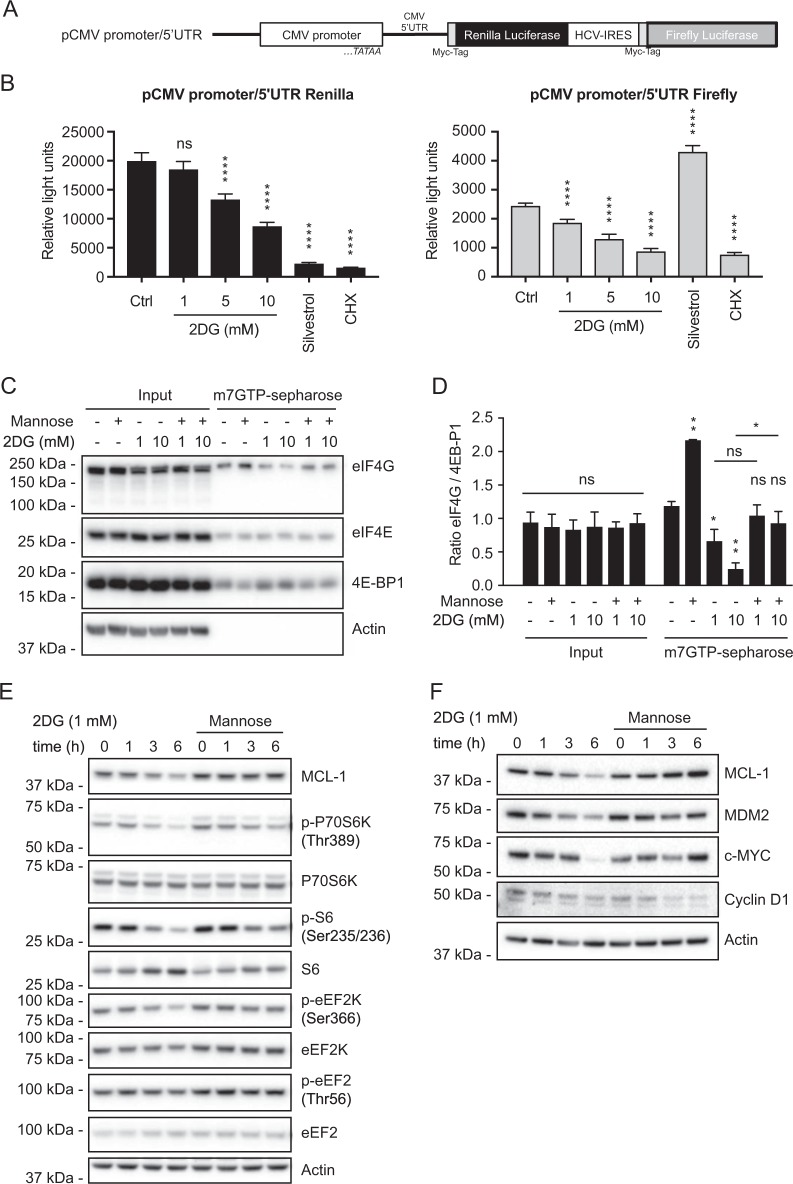
2DG perturbs two global protein synthesis pathways. **a** In the bicistronic reporter, the cap-dependent 5′UTR of cytomegalovirus (CMV) drives synthesis of renilla luciferase, whereas the IRES of hepatitis C virus (HCV) allows cap-independent translation of firefly luciferase [35]. **b** NALM-6 cells, immediately after electroporation with the dual reporter in **a** (see Methods), were incubated with 0, 1, 5, or 10 mM 2DG for 6 h, or treated for 6 h with silvestrol (100 nM) to inhibit cap-dependent initiation of translation or cycloheximide (CHX, 20μg/mL) to inhibit polypeptide elongation. Luminescence from renilla or firefly luciferase (see Methods) is plotted as mean ± SD (*n* = 3). Significance was determined by one-way ANOVA: ****P < 0.0001. Renilla luciferase clearly reflected cap-dependent initiation, because silvestrol, which inhibits initiation by interfering with RNA helicase eIF4A [21], abolished renilla but not firefly luciferase, whereas elongation-inhibitor cycloheximide markedly reduced both. The elevation in firefly luciferase by silvestrol is consistent with considerable evidence that marked suppression of cap-dependent translation boosts IRES-driven translation [34]. **c** Impact of 2DG on complexes with cap-binder eIF4E, analyzed by pull-down on m7GTP-sepharose beads, which bind eIF4E. Protein extracts from NALM-6 cells treated 6 h with 1 or 10 mM 2DG, with or without 10 mM mannose, were incubated with m7GTP-sepharose beads. Bound proteins were denatured and subjected to western blotting to reveal cap-associated eIF4E bound to eIF4G (active complexes) or to 4E-BP1 (inactive complexes). A representative blot is shown (*n* = 3). **d** The ratio of eIF4G/4E-BP-1 bound to eIF4E was quantified from three independent experiments like that in **c**, analyzed with ImageJ software. Data are plotted as mean ± SD. Significance of the difference from the untreated control (lane 7) determined by one-way ANOVA: *P < 0.05, **P < 0.01, or ns. **e** Western blot analysis of several pathways that influence the initiation and elongation stages of translation. The proteins analyzed here and in **d** affect formation of complexes critical for cap-dependent initiation (p70S6K, S6, eIF4E, eIF4G, 4E-BP1) or control of elongation (eEF2K and eEF2). **f** The levels of some short-lived proteins are affected more than others by the capacity of 2DG to inhibit global protein synthesis. Western blot analysis reveals that 2DG downregulates the short-lived oncoproteins MDM2 and c-MYC, but not cyclin D1. Supplementary Fig. 6 shows others unaffected by 2DG

Translation is predominantly regulated at its initiation, which typically requires engagement of the mRNA m7GpppN cap [21], but a mRNA sub-population may instead be translated cap-independently, e.g., by using an internal ribosome entry site (IRES), as in many viruses [34]. To determine which translation mode 2DG impaired, we exploited a bicistronic reporter in which the cap-dependent CMV promoter drives translation of renilla luciferase, whereas an IRES drives that of firefly luciferase [35] (Fig. 5a).

Both 5 and 10 mM 2DG significantly reduced renilla luciferase, 10 mM by 57% (Fig. 5b). Thus, 2DG markedly depresses cap-dependent translation. Unexpectedly, firefly luciferase also dropped at 1, 5, and 10 mM 2DG, falling ~ 65% at the highest dose. Hence, 2DG also markedly attenuates either elongation, like cycloheximide, or cap-independent initiation of translation (see below).

### 2DG reduces active complexes with cap-binder eIF4E

To confirm that 2DG impairs cap-dependent translation, we focused on its rate-limiting regulator, cap-binder eIF4E [21], which either recruits the mRNA into complexes with scaffold eIF4G that initiate translation or forms inactive complexes with 4E binding proteins (4E-BPs), which compete with eIF4G for binding eIF4E. Pull-down of eIF4E on cap-mimic m7GTP-Sepharose revealed that 2DG lowered the eIF4G bound to eIF4E but raised the 4E-BP1 (Fig. 5c). Indeed, the eIF4G/4E-BP1 ratio on the eIF4E bound to the cap-mimic declined ~ 45% with 1 mM 2DG and 79% with 10 mM 2DG (Fig. 5d). Thus, 2DG diminishes cap-dependent initiation at least partly by reducing active cap-binding complexes; presumably, mTORC1-mediated phosphorylation of 4E-BPs forces them to release eIF4E to bind eIF4G [21]. Interestingly, mannose precluded the drop in active complexes caused by 10 mM 2DG, but possibly independent of 2DG, because mannose also elevated the ratio without 2DG (compare lanes 7 and 8, Fig. 5d).

### 2DG alters signaling via key protein synthesis regulators

We then explored how 2DG reduced global translation. In accord with mTORC1 involvement (Fig. 5c, d), 2DG for 3 h consistently reduced phosphorylation of ribosomal protein S6 on Ser235/236 and of the responsible kinase, P70S6K on Thr389, while leaving their protein levels unchanged (Fig. 5e and Supplementary Fig. S3). Their diminished phosphorylation marks lower global translation and is ascribed to inhibition of mTORC1, the master regulator of protein synthesis [21, 36]. Indeed, p70S6K is inhibited by 2DG through mTORC1, because mTORC1 inhibitors torin-1 and rapamycin, similarly to 2DG, ablated phosphorylation of p70S6K and hence of its substrate S6 (Supplementary Fig. S4).

The eEF2 kinase regulates polypeptide elongation [37]. Its phosphorylation on Ser366, e.g., by AMPKα or by S6K downstream of mTORC1, inhibits its ability to phosphorylate its primary target eEF2 on Thr56, which slows elongation by reducing eEF2 activity [37]. After 3–6 h of 2DG treatment, eEF2K phosphorylation on Ser366 decreased (Fig. 5e and Supplementary Fig. 3), but as we have not observed increased phosphorylation of eEF2 on T56 (Fig. 5e), the significance of the reduced eEF2K phosphorylation remains uncertain (see Discussion).

To assess whether 2DG acts through common pathways in other hematopoietic tumors, we immunoblotted proteins from 2DG-treated JURKAT and SUDHL-4 cells with the antibodies that had revealed altered regulation of NALM-6 protein synthesis (Supplementary Fig. S5). MCL-1 dropped markedly in both JURKAT and SUDHL-4, as did phosphorylated P70S6K and S6, indicating reduced mTORC1 activity, and eEF2K phosphorylation fell, but again its primary substrate eEF2 appeared unaffected. Mannose co-treatment attenuated all these changes. However, ATF4 upregulation was just detectable in SUDHL-4 and absent from JURKAT. Hence, these three lines respond similarly albeit not identically.

### Certain proteins with short half-lives are particularly susceptible to 2DG downregulation

The marked drop in MCL-1 by 2DG must reflect not only the reduced global translation but also the short half-life of MCL-1 protein. We, therefore, checked how 2DG affected other short-lived proteins. Indeed, it lowered the important oncoproteins c-MYC and MDM2 (Fig. 5f), which regulates the major tumor suppressor p53. However, cyclin D1 was unaffected, as were seven other short-lived proteins: IκBα, CDC25C, cyclin E, cyclin A, p53, XIAP, and c-IAP1 (Supplementary Fig. S6). Hence, factors in addition to protein half-life must affect the translation efficiency of mRNAs.

### The structured 5′UTR of *MCL-1* mRNA can attenuate its translation

**Fig. 6:**
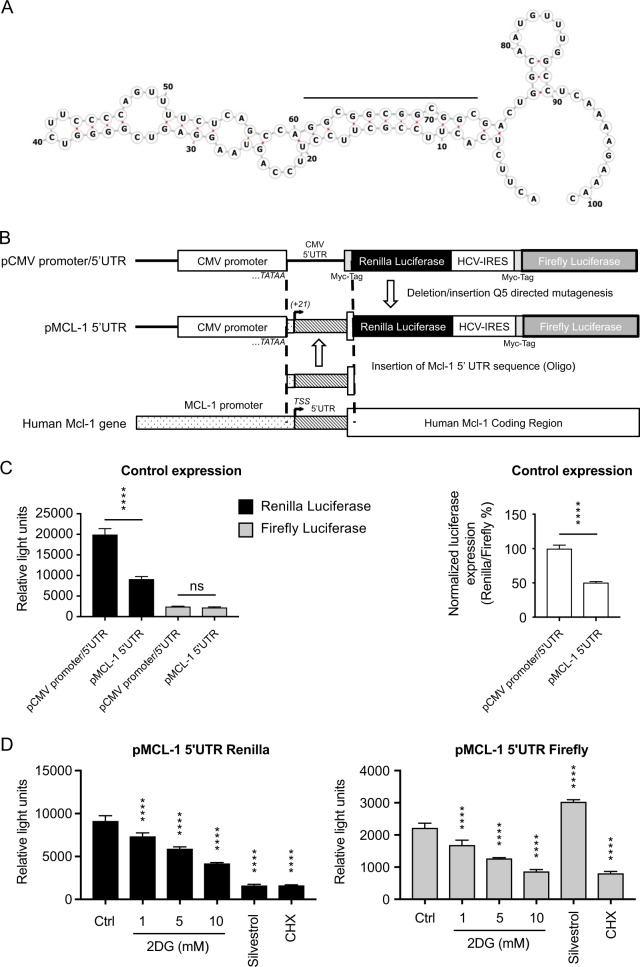
Structure in the 5′UTR of *MCL-1* mRNA affects its translation efficiency. **a** The minimal-free energy (MFE) structure for the 80-nucleotide *MCL-*1 5′UTR and the first 21 nucleotides of its coding region, as assigned by the Vienna RNA Websuite. The optimal MFE structure shown is −26.4 kcal/mol, while the free energy of the thermodynamic ensemble of predicted structures is −28.55 kcal/mol. **b** In the dual reporter vector pMCL-1 5′UTR, the 5′UTR of the CMV major immediate early region mRNAs, between the CMV promoter and the renilla luciferase coding region, is replaced by the full 80 bp of the *MCL-1* 5′ UTR plus the first 13 nucleotides of the *MCL-1* coding region, since the latter contributes to the predicted secondary structure in **a**. **c** Efficiency of the *MCL-1* 5′UTR compared with that of CMV in producing renilla luciferase and firefly luciferase as a control, in the absence of 2DG. NALM-6 cells were electroporated with the two reporter constructs and the levels of the two luciferases determined 6 h later (see Fig. 5b and Methods). The *MCL-1* 5′UTR drove renilla luciferase only half as efficiently as the CMV 5′UTR (*n* = 3), whether the raw data was compared (left panel), or the renilla data was normalized to that obtained with firefly luciferase (right panel), to preclude any differences due to electroporation efficiency or transcription. In fact, the firefly results for the two constructs did not differ significantly. **d** Reduction by 2DG in translation of renilla luciferase driven by the *MCL-1* 5′UTR. As in **c**, NALM-6 cells electroporated with the *MCL-*1 5′UTR were treated with 1, 5, or 10 mM 2DG for 6 h and the two luciferases then assayed (see Methods). The % reductions in translation by 2DG (*n* = 3) are comparable to those with the CMV 5′UTR (Fig. 5b) but highly significant even with 1 mM 2DG

A long 5′UTR with secondary structure is thought to render mRNAs more dependent on eIF4F [21–23]. Although the 5′UTR of *MCL-1* mRNA is only moderately long (80 nucleotides vs. a median of 218 for human mRNAs [38], the well-regarded Vienna University RNA folding program (http://rna.tbi.univie.ac.at//cgi-bin/RNAWebSuite/RNAfold.cgi) predicts substantial secondary structure (Fig. 6a).

To determine whether the *MCL-1* 5′UTR can attenuate translation, we replaced the CMV 5′UTR in the Fig. 5a reporter with that of *MCL-1* (Fig. 6b). In the absence of 2DG (Fig. 6c), the *MCL-1* 5′UTR drove only 45% as much renilla luciferase as the CMV 5′UTR, suggesting that *MCL-1* 5′UTR sequences may indeed influence translation. Moreover, as in Fig. 5b, 2DG further lowered renilla luciferase, 10 mM 2DG evoking an additional 44% reduction (Fig. 6d, left panel). Thus, the structured *MCL-1* 5′UTR likely contributes to the 2DG-induced dearth of MCL-1 protein. As expected, the firefly luciferase levels (Fig. 6d, right panel) mirrored those in Fig. 5b, re-enforcing our conclusion that 2DG also impairs elongation and/or cap-independent translation.

## Discussion

Our results support the view that the augmented killing of many tumor cells co-treated with 2DG and a BH3 mimetic like ABT737 [9–11] largely reflects the 2DG-induced drop in MCL-1. Pro-survival relatives did not decline, and none of nine pro-apoptotic relatives increased (Fig. 3a). The latter might seem surprising, since BIM and PUMA mediate, in some cells, the death from protracted ER stress [29, 30]. However, 2DG did not induce a robust complete ER stress response in NALM-6 cells (Fig. 2), perhaps because its PERK/eIF2α/ATF4/CHOP arm attenuates the pro-survival IRE1/XBP1 arm to favor apoptosis [39]. Also, lymphoid tumor cell death driven by 2DG plus ABT737did not require BIM, PUMA, or NOXA [11]. Moreover, although NOXA can promote MCL-1 degradation, NOXA-independent routes are common [32].

**Fig. 7:**
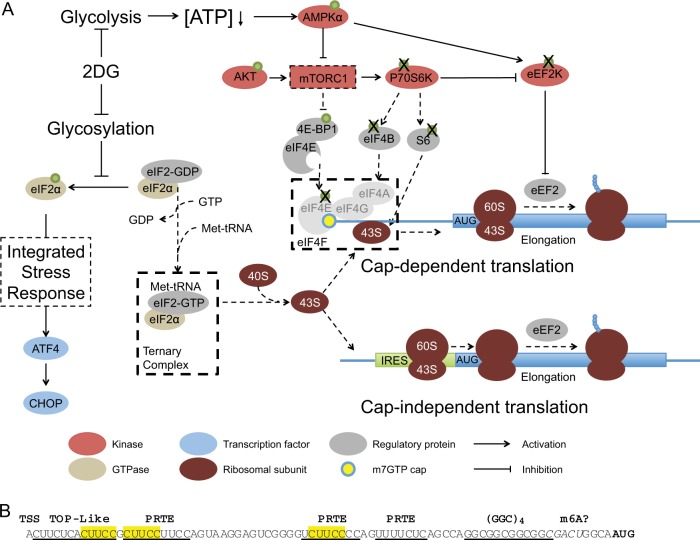
Model for the impact of 2DG on global mRNA translation and sequence motifs in the *MCL-1* 5′UTR. **a** The model integrates the present results with current understanding of translational regulation [21–23]. Our findings suggest that 2DG impedes global translation in several ways, primarily by reducing both glycolysis and protein glycosylation in the ER. By reducing glycolysis (top), 2DG rapidly reduces ATP, transiently activating AMPKα, which can (directly or indirectly) inhibit the mTORC1 complex. Downregulated mTORC1 signaling reduces phosphorylation of ribosomal protein S6, a mark of active translation, and of its kinase p70S6K, as well as 4E-BP1, which prevents association of eIF4E with eIF4G until phosphorylated by mTORC1. Thus, the reduced mTORC1 signaling reduces assembly of the eIF4F complex, limiting the initiation of cap-dependent translation, and may lead to inhibition of eEF2K, which might then attenuate polypeptide elongation. The perturbed N-linked glycosylation in the ER by 2DG (left side) produces an ISR triggered by increased phosphorylation of eIF2α, which reduces the level of the ternary complex (GTP-eIF2 plus Met_i_-tRNA). This limits recruitment of both Met_i_-tRNA and the small ribosomal subunit to the mRNA, impairing translation of most mRNAs but switching on translation of the stress-regulated transcription factors ATF4 and CHOP and other proteins needed for homeostasis. Notably, 2DG impairs not only cap-dependent translation but also cap-independent translation (Figs. 5b and 6d), such as that driven by an IRES, e.g., in *MYC* or *MDM2* mRNA (Fig. 5f). **b** Sequence motifs in the *MCL-1* 5′UTR. The transcriptional start site (TSS) is followed by a TOP-like sequence. Whereas TOP mRNAs have a C just after the cap, followed by 4–15 pyrimidines, a TOP-like mRNA does not start with a C and the pyrimidine run can start a few residues later. The three pyrimidine runs, denoted pyrimidine-rich translational elements (PRTEs), include two with a U at position six, as found frequently in mTORC1-sensitive mRNAs; these sequences resemble some of those in the 5′UTR of TOP mRNAs. The MCL-1 5'UTR also contains a (GGC)_4_ sequence and a near consensus m6A methylation site

Notably, 2DG markedly reduced global polypeptide synthesis (Fig. 4d). As Fig. 7a outlines, 2DG, probably by inhibiting N-linked glycosylation in the ER, provoked an ISR involving eIF2α phosphorylation, perhaps by PKR-like ER kinase (PERK). This shuts down cap-dependent translation and activates selective translation of transcripts that can restore homeostasis, e.g., transcription factor ATF4 and its target CHOP [17, 18, 25, 26] (Fig. 2). Phosphorylated eIF2α inhibits GDP/GTP exchange by eIF2B, lowering the ternary complex (GTP-bound eIF2α plus the loaded initiator Met-tRNA) (Fig. 7a). Since this complex conveys both the small ribosomal subunit and Met-tRNA to mRNAs [25, 26], global translation initiation falls, probably on both cap-independent and cap-dependent mRNAs (Fig. 7a).

Cap-dependent translation also requires assembly on the mRNA cap of the eIF4F complex, comprising cap-binder eIF4E, scaffold eIF4G and helicase eIF4A, which unwinds 5′UTR secondary structure (Fig. 7a) [21–23]. Since the proportion of eIF4E in active complexes with eIF4G fell ~ 80% with 10 mM 2DG (Fig. 5d), 2DG reduces both the complexes critical for cap-dependent translation: the ternary complex and eIF4F (Fig. 7a). Full MCL-1 reduction may require lowering both complexes, because mTORC1 inhibitors ablated phosphorylation of key mTORC1 substrate p70S6K but did not notably diminish MCL-1 (Supplementary Fig. S4). Thus, full 2DG cytotoxicity probably requires both the ISR and reduced mTORC1 signaling (Fig. 7a). The reduction in 2DG-induced cytotoxicity by mannose has sometimes been ascribed primarily to relief of 2DG-provoked ER stress [12, 17, 18], but, contrary to findings with some tumor lines [17, 18, 40], mannose also attenuated the 2DG-induced ATP drop (Fig. 4a), consistent with critical roles for both the AMPK/mTORC1 and ER ISR pathways (Fig. 7a).

Figure 7a illustrates how reduced glycolysis by 2DG probably attenuates eIF4F formation. The rapid ATP drop (Fig. 4a), the earliest change we observed, transiently activated AMPKα (Fig. 4b), which then inhibits mTORC1, the driver of translation [21–23]. Lower mTORC1 activity reduces 4E-BP phosphorylation, leaving more eIF4E in inactive 4E-BP complexes than active eIF4G ones (Fig. 5d). Although 2DG inactivates mTORC1, we have not observed consistent phosphorylation changes on mTORC1 components, probably because its activity more critically depends upon protein association and lysozyme membrane localization [41], as does AMPK activity [33].

Lower mTORC1 activity also reduces phosphorylation (activation) of p70S6K and hence of its substrate ribosomal protein S6 (Fig. 5e and Supplementary Fig. S3), as mTORC1 inhibitors showed (Supplementary Fig. S4). Although active P70S6K can increase cap-dependent translation by phosphorylating eIF4B, augmenting eIF4A helicase activity, 2DG has not consistently reduced eIF4B, so mTORC1 may more commonly control eIF4E via 4E-BPs.

Notably, dual reporters revealed that 2DG impaired not only cap-dependent translation, but also elongation and/or cap-independent initiation (Figs. 5b and 6d). Activated AMPKα can phosphorylate and activate elongation regulator eEF2K [37] (Fig. 7a), allowing eEF2K to phosphorylate its primary substrate eEF2 on Thr56, inactivating eEF2 and attenuating polypeptide elongation, as observed in nutrient-deprived transformed cells [42]. Although 2DG did modestly reduce eEF2K phosphorylation on an activating site (Ser366) (Fig. 5e and Supplementary Fig. S3), the absence of increased eEF2 phosphorylation on Thr56 argues against reduced elongation via eEF2, unless eEF2K can inhibit eEF2 in another way, or act via another substrate [43].

Alternatively, the firefly luciferase results (Figs. 5b and 6d) may reflect reduced translation by 2DG of mRNAs using cap-independent initiation. Although controversial, an IRES may drive translation of 10% of mammalian mRNAs [34], including mRNAs crucial for cell survival (e.g., BCL-XL, BCL-2, XIAP, cIAP1), proliferation (IGF2, MYC) or the cell cycle (p27, p53, MDM2) [34]. Pertinently, 2DG reduced MYC and MDM2 levels (Fig. 5f). However, 2DG might also or instead impair initiation mechanisms not requiring an IRES or cap [34, 44], particularly engagement of N6-methyladenosine (m6A) in the 5′UTR [45–47]. Pertinently, the MCL-1 5′UTR contains a motif resembling the m6A consensus (Fig. 7b). A single m6A in a 5′UTR can recruit eIF3 and initiate translation independent of the cap and eIF4F [45–47].

The marked MCL-1 decrease by 2DG must reflect not only impaired global translation but also the short half-life of MCL-1 protein. Although 2DG did downregulate two other short-lived oncoproteins, c-MYC and p53 regulator MDM2 (Fig. 5f), eight other short-lived proteins were unaffected (Supplementary Fig. S6). Hence, additional factors must determine which mRNAs are most downregulated by 2DG.

A structured 5′UTR, like that predicted for MCL-1 (Fig. 6a), is thought to reduce translation frequency and render mRNAs dependent upon eIF4F [38]. Pertinently, MCL-1 translation requires both cap-binder eIF4E and helicase eIF4A [48]. Notably, the *MCL-1* 5′UTR drove reporter translation only half as efficiently as the CMV 5′UTR (Fig. 6c), and 2DG further reduced translation (Fig. 6d). Thus, its secondary structure likely contributes to the reduced MCL-1 protein. Notably, a (GGC)_4_ sequence in the *MCL-1* 5′UTR (Fig. 7b) resembles those greatly enriched in the 5′UTR of mRNAs requiring helicase eIF4A [49, 50]. Although such sequences were proposed to form G-quadruplexes [49, 50], single (GGC)_4_ sequences are now thought to instead form Watson-Crick secondary structures like that in Fig. 6a [51].

Linear motifs within 5′UTRs also affect translation frequency. The *MCL-1* 5′UTR contains short pyrimidine-rich motifs like those enriched in mRNAs whose translation requires eIF4F (Fig. 7b). A ‘‘TOP-like sequence” [52], i.e., one resembling the 5′-Terminal Oligo-Pyrimidine (TOP) sequences on mRNAs encoding ribosomal proteins and translation factors [53, 54], is followed by other pyrimidine-rich translational elements (PRTEs) [55], which mark mRNAs controlled by mTORC1. Interestingly, the PRTEs in the *MCL-1* 5′UTR resemble some in TOP mRNAs engaged by the RNA- and cap-binding protein LARP1, a mTORC1 target that controls TOP mRNA translation [56–59]. LARP1 competes with eIF4E for cap-binding and prevents TOP mRNA translation until mTORC1 phosphorylates LARP1 to free them [57].

Intriguingly, several *MCL-1* 5′UTR PRTE sequences contain CUUCC (Fig. 7b), which occurs near the 5′-end of certain TOP mRNAs, e.g., eEF2, rpL32, and eIF3A [53, 54]. Hence, we suggest that MCL-1 translation may be regulated analogously: an RNA-binding protein that engages PRTE sites, and perhaps also the cap, might sequester *MCL-1* mRNA until phosphorylated by mTORC1. The regulator might be LARP1 or its little-studied close relative LARP2 (LARP1B), which contains the DM15 domain by which LARP1 binds the cap and oligo-pyrimidine track [57]. Its affinity for 5′UTR PRTE motifs might determine which mRNAs 2DG strongly downregulates.

In summary, 2DG may well lower MCL-1 protein by impeding global mRNA translation (Fig. 7a), if one considers also its 5′UTR structure and sequence motifs, which may restrict translation efficiency under stress (Figs 6 and 7b), and the short MCL-1 half-life. Nevertheless, reduced global translation must affect numerous proteins that influence 2DG-induced cytotoxicity, e.g., MDM2 and MYC (Fig. 5f). Although 2DG has potential for cancer treatment, its low potency may restrict its application. However, our results support the likelihood [21–23] that more potent and specific translation inhibitors will advance cancer therapy, particularly together with BH3 mimetics.

## Materials and methods

### Compounds, cell lines, and culture conditions

2-deoxy-d-glucose (2DG), mannose (Mn), thapsigargin (Thps), tunicamycin (Tun), brefeldine A (BFA), cycloheximide (CHX), and MG132 were from Sigma-Aldrich (St. Louis, MO, USA). Q-VD-OPh was from MP Biomedicals Australasia. Cell lines from human leukemias (NALM-6, HL-60, REH, RS4;11, and K562) or lymphomas (JURKAT, SUDHL-4, and RAJI) were cultured at 37 °C under 5% CO2 atmosphere in RPMI-1640 (Thermo Fisher Scientific, Waltham, MA, USA), supplemented with 10% fetal bovine serum (FBS) from Sigma-Aldrich and 2 mM GlutaMAX (Thermo Fisher Scientific). Unless otherwise indicated, cultures were maintained by seeding at 4 × 10^5^ to 1 × 10^6^ cells/mL twice a week.

### Cytofluorometric assessment of cell cycle and apoptosis

For cell cycle determination, 5 × 10^5^ cells were collected, washed once with ice-cold phosphate-buffered saline and permeabilized with 100 μL of citrate/PI buffer (0.1% Triton x-100, 50μg/mL propidium iodide, 0.1% sodium citrate) under vortex agitation. Cells were incubated 15 min on ice before cytofluorometric acquisition. To assess apoptosis, 5 × 10^5^ cells were collected, washed once with HBSS (Hank’s Balanced Salt Solution: 400 mg/L KCl, 60 mg/L KH_2_PO_4_, 350 mg/L sodium bicarbonate, 8 g/L NaCl, 48 g/L Na_2_HPO_4_, 1 g/L dextrose) supplemented with 10% FBS and resuspended in 100 μL of staining solution (HBSS, 10% FBS, Annexin-V-FITC) and incubated at 37 °C for 30 min. Propidium iodide was added (to 1μg/mL) prior to data acquisition on a FACScalibur cytofluorometer (BD Bioscience).

### Cytochrome c release from mitochondria

After drug treatment, 1 × 10^6^ cells were suspended in permeabilisation buffer (300 mM sucrose, 10 mM Tris/HCl pH 7.4, 1 mM EDTA, 0.025% digitonin with cOmplete™, Mini, EDTA-free protease inhibitor cocktail (Roche, Dee Why, NSW Australia)) and incubated on ice for 10 min. After centrifugation at 13,000 rpm for 5 min at 4 °C, the supernatant (cytosolic fraction) was saved while the pellet (mitochondrial fraction) was resuspended in mitochondrial lysis buffer (300 mM sucrose, 10 mM Tris/HCl pH 7.4, 1 mM EDTA, 1% digitonin with the protease inhibitors above). The cytosolic and mitochondrial fractions were denaturated in NuPAGE™ LDS Sample Buffer (Thermo Fisher Scientific) before analysis by western blotting.

### Western blotting and antibodies

Total cell lysates were prepared by lysing cells directly into ONYX buffer (20 mM Tris-HCl pH 7.4, 135 mM NaCl, 1.5 mM MgCl_2_, 1 mM EGTA, 1% nonyl phenoxylpolyethoxylethanol [NP-40], 10% glycerol), supplemented with the Roche protease inhibitor cocktail and Roche PhosSTOP™ phosphatase inhibitor cocktail. After protein quantification (DC Protein Assay, Bio-Rad), 20μg of protein was separated on 4–12% NUPAGE Bis Tris gels (Invitrogen), then transferred to polyvinylidene difluoride (PVDF) and blotted with various antibodies to human proteins.

The antibodies and their sources were: cytochrome c (556433, BD Pharmingen), BOK (kindly provided by Prof. Thomas Kaufmann, Bern University), BAD (ADI-AAP-020, Enzo Life Science), PUMA (3043, Sapphire Bioscience), NOXA (2437, ProSci Incorporated), BIM (ADI-AAP-330-E, Sapphire Bioscience), Actin (A2228, Sigma-Aldrich), MDM2 (sc-812, Santa Cruz Biotechnology), c-Myc (sc-764, Santa Cruz Biotechnology). The Walter and Eliza Hall Antibody Facility made the following antibodies: MCl-1 (19C4–15), BCL-2 (Bcl-2-100), BCL-X_L_ (9C9), BAK (7D10), BAX (21C10-23-8-38-P), BMF (12E10), BID (2D1-3). The following antibodies were from Cell Signalling Technology (Danvers, MA, USA): PARP (#9532), p-eIF2α Ser51 (#3597), eIF2α (#5324), ATF4 (#11815), CHOP (#2895), XBP1s (#12782), ATF6 (#65880), BIP (#3183), p-AMPKα Thr172 (#2535), AMPKα (#2532), p-ACC Ser79 (#11818), ACC (#3676), p-AKT Ser473 (#4060), AKT (#4691), p-P70S6K Thr389 (#9234), P70S6K (#2708), p-S6 Ser235/236 (#4858), S6 (#2217), p-eEF2K Ser366 (#3691), eEF2K (#3692), p-eEF2 Thr56 (#2331), eEF2 (#2332), eIF4G (#2469), eIF4E (#2013), 4E-BP1 (#9644), cyclin D1 (#2978).

Secondary anti-Rat/Mouse/Rabbit IgG antibodies conjugated to HRP were from Southern BioTech (Birmingham, AL, USA). Luminescence was determined on a ChemiDoc XRS + machine with ImageLab software (Bio-Rad) using a Luminata Forte Western HRP substrate (Millipore, Billerica, MA, USA).

### Real-time qPCR analyses and RT-PCR for XBP1

Total RNA was isolated from cells using TRIzol (Thermo Fisher Scientific). The RNA pellet was washed and treated 1 h with DNase (Turbo DNA-Free, Thermo Fisher Scientific) and RNA then quantified (Themo Scientific Nanodop 1000). Reverse transcription of 2μg of RNA was performed, using High-Capacity cDNA Reverse Transcription Kits (Applied Biosystems) with oligo-d(T) primer, and 10 ng of the cDNA was then subjected to real-time qPCR in triplicate (SYBR Select Master Mix, Applied Biosystems) on a ViiA7 Real-time apparatus (Thermo Fisher Scientific). The oligonucleotide pairs used for qPCR were as follows: MCL-1 (forward 5′-CATTCCTGATGCCACCTTCT-3′, reverse 5′-TCGTAAGGACAAAACGGGAC-3′); and housekeeping mRNAs GAPDH (forward 5′-AAGGTGAAGGTCGGAGTCAA-3′, reverse 5′-AATGAAGGGGTCATTGATGG-3′), TUBULIN-B1 (forward 5′-TCGATGCCATGTTCATCACT-3′, reverse 5′- TAACCATGAGGGAAATCGTG-3′), PPIA (Peptidylprolyl Isomerase A, forward 5′-CACCGTGTTCTTCGACATTG-3′, reverse 5′-TTCTGCTGTCTTTGGGACCT-3′) and TBP (TATA-box-binding protein, forward 5′-AACAACAGCCTGCCACCTTA-3′, reverse 5′-GCCATAAGGCATCATTGGAC-3′).

The oligonucleotide pairs used for RT-PCR on human XBP1 were as follows: 5′-CCTGGTTGCTGAAGAGGAGG-3′ and 5′-CCATGGGGAGATGTTCTGGAG-3′. PCR products were analyzed on a 3.5% agarose gel on ChemiDoc XRS machine with ImageLab software (Bio-Rad).

### ATP quantification

ATP was quantified using a Luminescent ATP Detection Assay Kit (ab113849, ABCAM). Briefly, 2 × 10^4^ NALM-6 cells were seeded in triplicate in a white 96-well plate. They were treated with 2DG (1 or 10 mM), with or without mannose (10 mM), for 0.5, 1, 3, and 6 h. After cell lysis with the detergent solution provided, D-luciferin and luciferase were added, and 10 min later, luminescence was measured with a Hidex Chameleon plate reader. The light emitted during the reaction is proportional to the ATP present.

### m7GTP binding assay for eIF4E complexes

Cells were homogenized in lysis buffer (20 mM Tris-HCl pH 7.4, 135 mM NaCl, 1.5 mM MgCl_2_, 1 mM EGTA, 1% nonyl phenoxylpolyethoxylethanol (NP-40) and 10% glycerol), supplemented with the Roche protease inhibitor and Roche PhosSTOP™ phosphatase inhibitor cocktails. Cell extracts (100μg protein) were incubated with 20 μL of m7GTP-sepharose beads (AC-155, Jana Bioscience, Germany) for 30 min in a rotary suspension mixer at 4 °C. The beads were washed twice with lysis buffer, then the bound proteins were denatured by 5 min at 95 °C in NuPAGE™ LDS Sample Buffer. After a brief centrifugation, supernatants were electrophoresed on a NUPAGE gel, and the proteins transferred onto a PVDF membrane and immunoblotted.

### Labeling and quantifying newly synthesized protein

To label newly synthesized protein, 30 min before the end of cell treatment, 100 μCi of ^35^S-methionine/^35^S-cysteine was added per mL of cell culture (EXPRE^35^S^35^S Protein Labeling Mix, Perkin Elmer). After removing media and washing the cell pellets twice with phosphate-buffered saline by centrifugation, cells were lysed in RIPA buffer (50 mM Tris/HCl pH 8.0, 150 mM NaCl, 1% NP-40, 0.5% sodium deoxycholate, 0.1% sodium dodecyl sulfate (SDS), supplemented with the Roche protease and phosphatase inhibitor cocktails above. After protein quantification (DC Protein Assay, Bio-Rad), 50μg of protein was spotted on Whatman paper pre-blocked with an amino acid mixture (11130, Thermo Fisher Scientific). To precipitate polypeptides with trichloroacetic acid (TCA), the dried paper was exposed to 10% TCA + 0.1% methionine at 4 °C for 20 min, boiled in 5% TCA for 15 min, washed with 5% TCA, and then with ethanol. Finally, the dried paper was subjected to scintillation counting in a Hidex 300 SL apparatus. Also, 20μg of the labeled protein was denatured by 5 min at 95 °C with NuPAGE™ LDS Sample Buffer (Thermo Fisher Scientific), resolved by polyacrylamide gel electrophoresis, and transferred onto a PVDF membrane, which was dried and scintillations detected on a Typhoon FLA 7000 machine (GE Life Science).

### Reporter constructs and their expression

The pMCL-1 5′UTR construct (see Fig. 6b) was derived from the pCDNA3/Ren/HCV/FF plasmid [60], kindly provided by Prof. Pelletier and Dr. Robert, using the *Q5*® Site-Directed Mutagenesis Kit (#E0552S, New England Biolabs), following their substitution protocol. Oligonucleotides spanning the human *MCL-1* 5′UTR sequence, including the first 13 nucleotides of the MCL-1 coding sequence, and relevant mutagenesis primers were purchased from Bioneer Pacific (East Kew, Victoria, Australia). Sequencing confirmed correct assembly. In both vectors, the ATG initiating renilla luciferase has a good Kozak context with a purine at minus 3. Five million NALM-6 cells were electroporated with 5μg of the constructs using the AMAXA Nucleofector kit V program T-01 (Lonza, Cologne Germany), which gave high incorporation and cell viability. Immediately after electroporation, the cells were resuspended in 2 mL of fresh medium and 50 μL aliquots seeded in quadruplicate into a white 96-well plate and treated (or not) with 2DG for 6 h. Renilla and firefly luciferases were quantified on a Hidex Chameleon plate reader following the instructions with the Dual-Glo Luciferase Assay System (#TM058, Promega).

### Statistical analysis

All statistical tests were performed using Prism 7 (GraphPad, La Jolla, CA, USA). Two-group comparisons were made using Student′s *t*-test assuming equal variances. Multiple groups were analyzed, as indicated, by either one-way or two-way ANOVA with Turkey′s multiple comparisons tests. Unless otherwise indicated, all data are presented as mean ± SD with a significant *P*-value (*P < 0.05, **P < 0.01, ***P < 0.001, ****P < 0.0001, or ns for not significant)

## Electronic supplementary material

Supplementary Figure 1

Supplementary Figure 2

Supplementary Figure 3

Supplementary Figure 4

Supplementary Figure 5

Supplementary Figure 6

supplementary figure legends
