# Supplementary figures and images for: By reducing global mRNA translation in several ways, 2-deoxyglucose lowers MCL-1 protein and sensitizes hemopoietic tumor cells to BH3 mimetic ABT737
Source: Cell Death Differ. 2018 Dec 11;26(9):1766–81. doi: 10.1038/s41418-018-0244-y (PMC6748140; doi:10.1038/s41418-018-0244-y)

Supplementary Fig 1

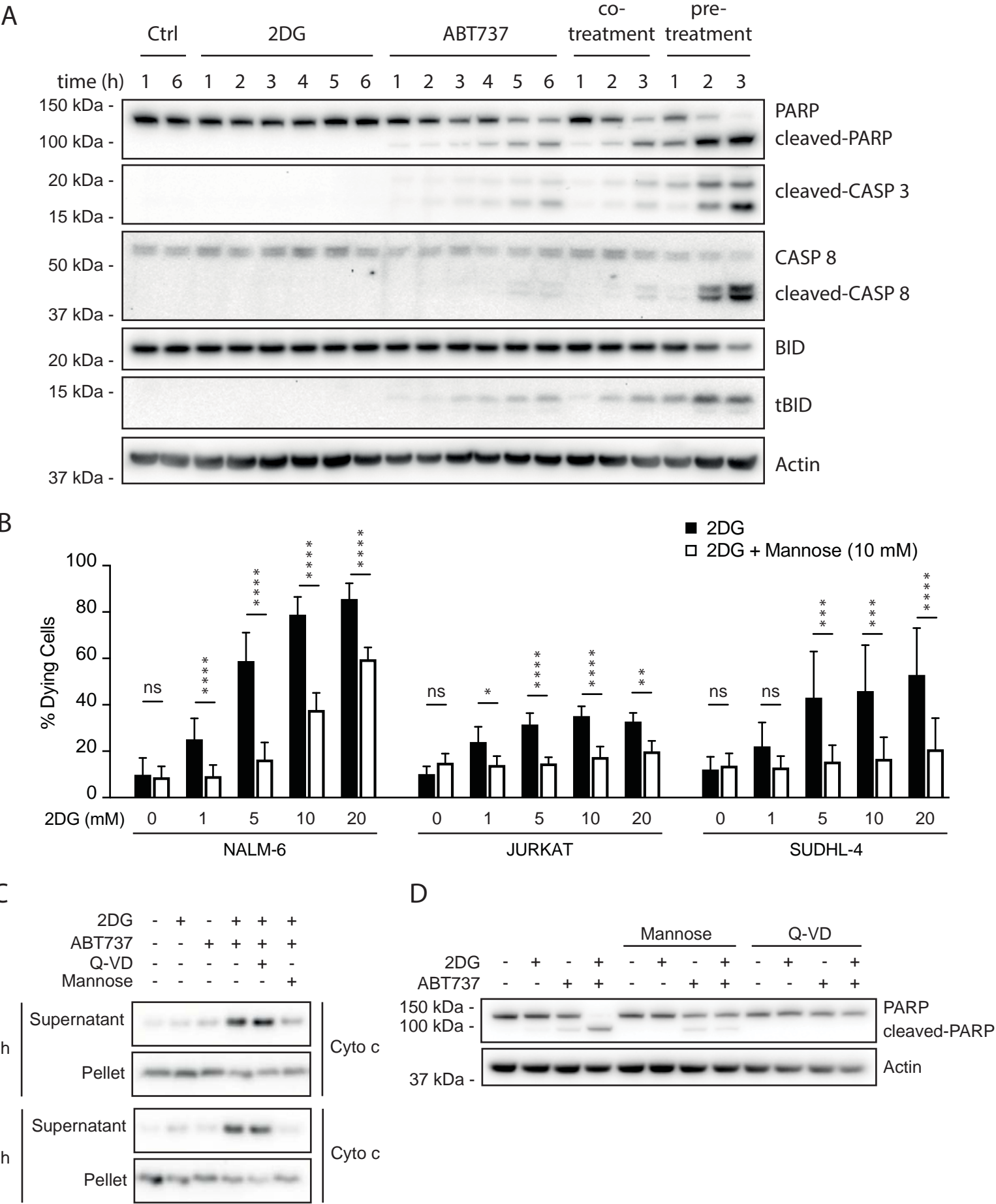

Supplement: Supplementary file 1 — Supplementary Figure 1 [file 41418_2018_244_MOESM1_ESM.pdf]

Supplementary Fig 2

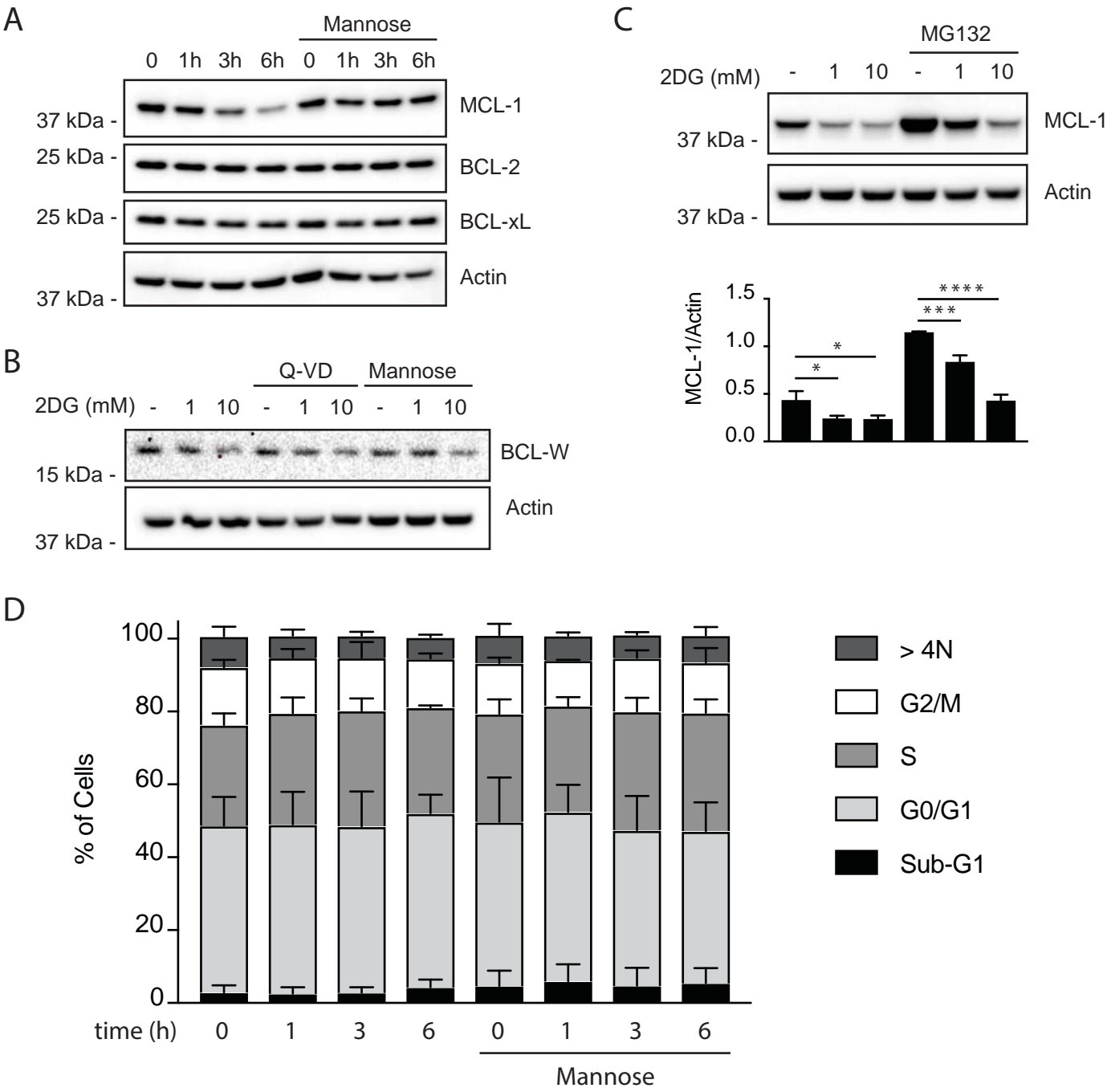

Supplement: Supplementary file 2 — Supplementary Figure 2 [file 41418_2018_244_MOESM2_ESM.pdf]

Supplementary Fig 3

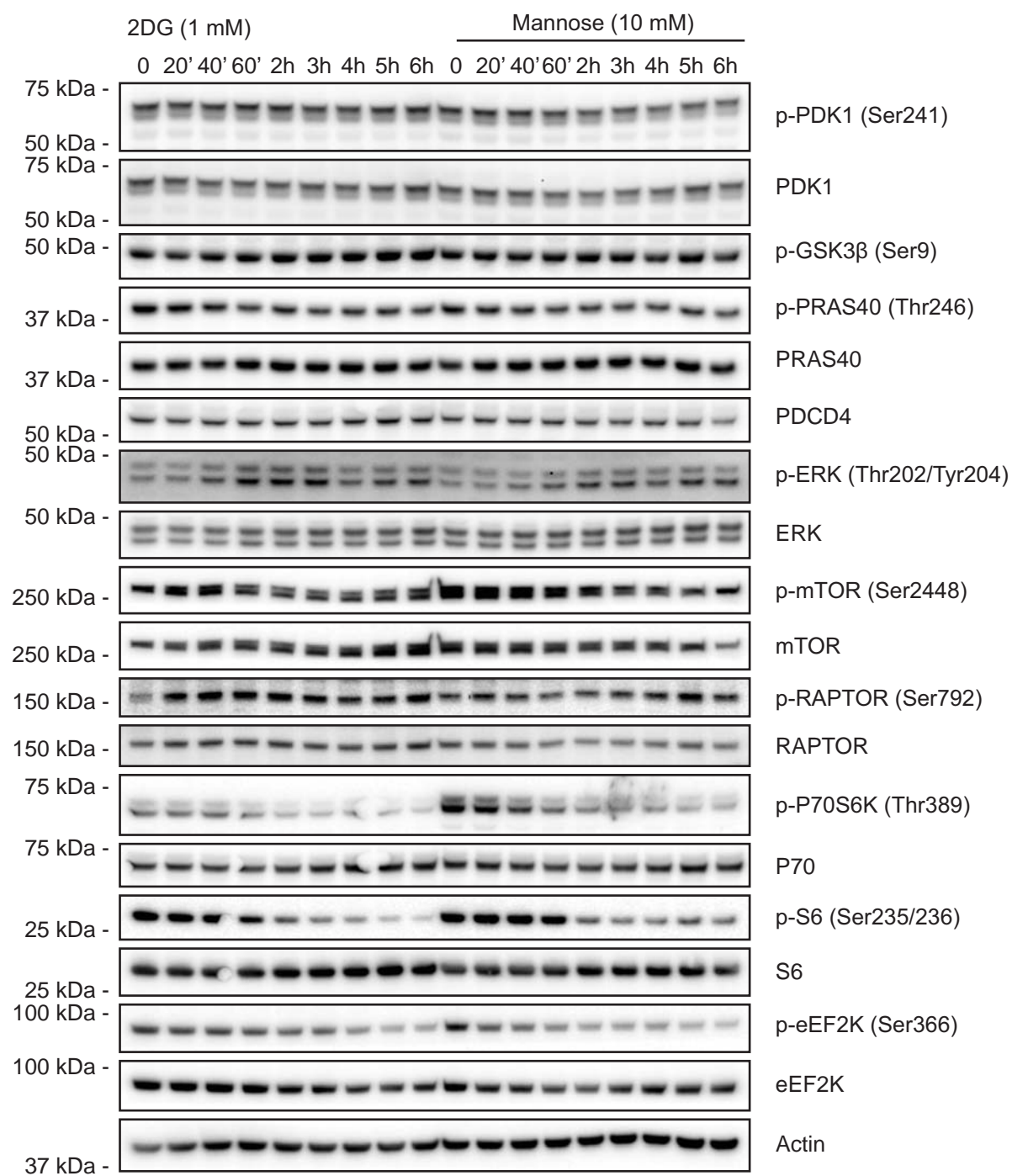

Supplement: Supplementary file 3 — Supplementary Figure 3 [file 41418_2018_244_MOESM3_ESM.pdf]

Supplementary Fig 4

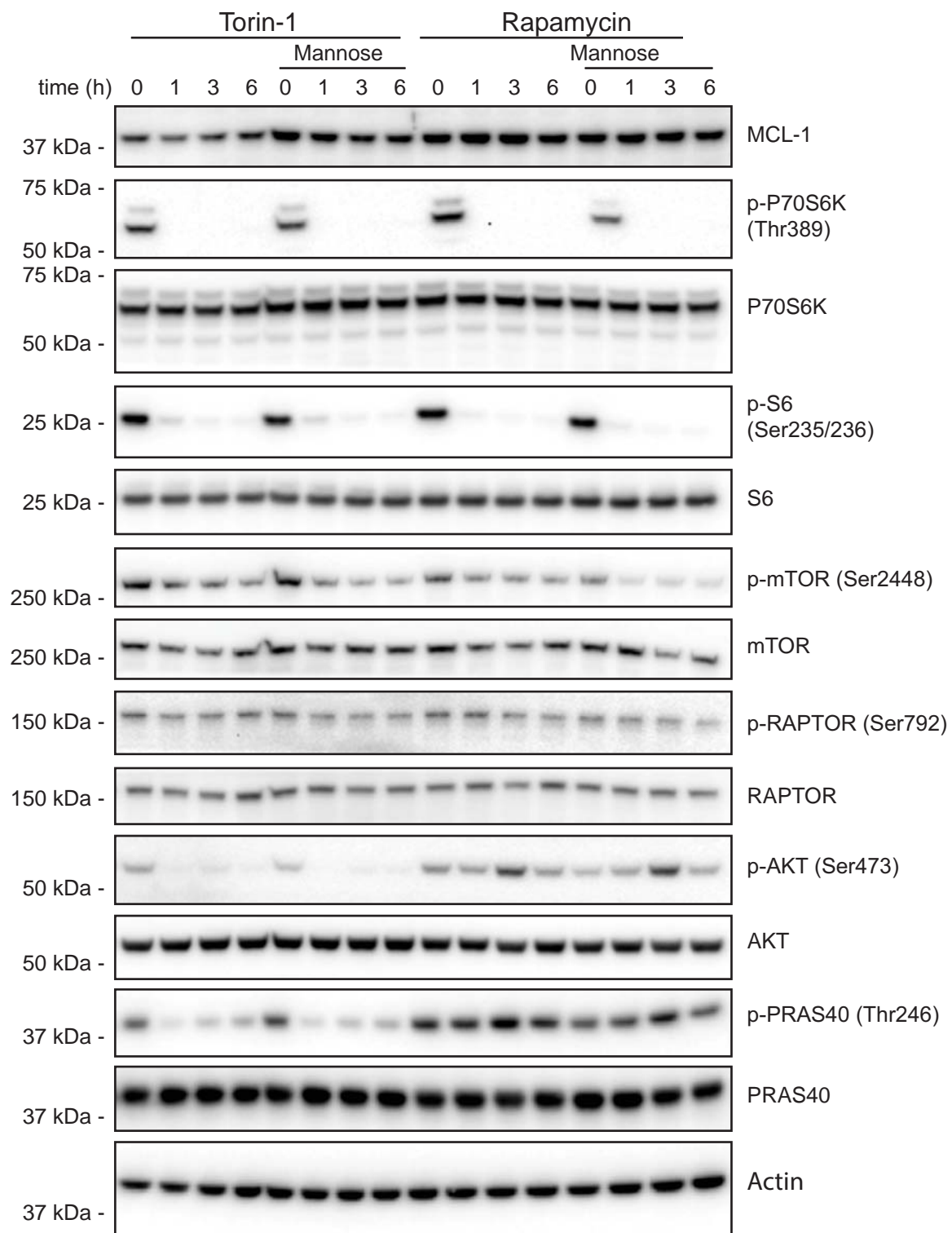

Supplement: Supplementary file 4 — Supplementary Figure 4 [file 41418_2018_244_MOESM4_ESM.pdf]

Supplementary Fig 5

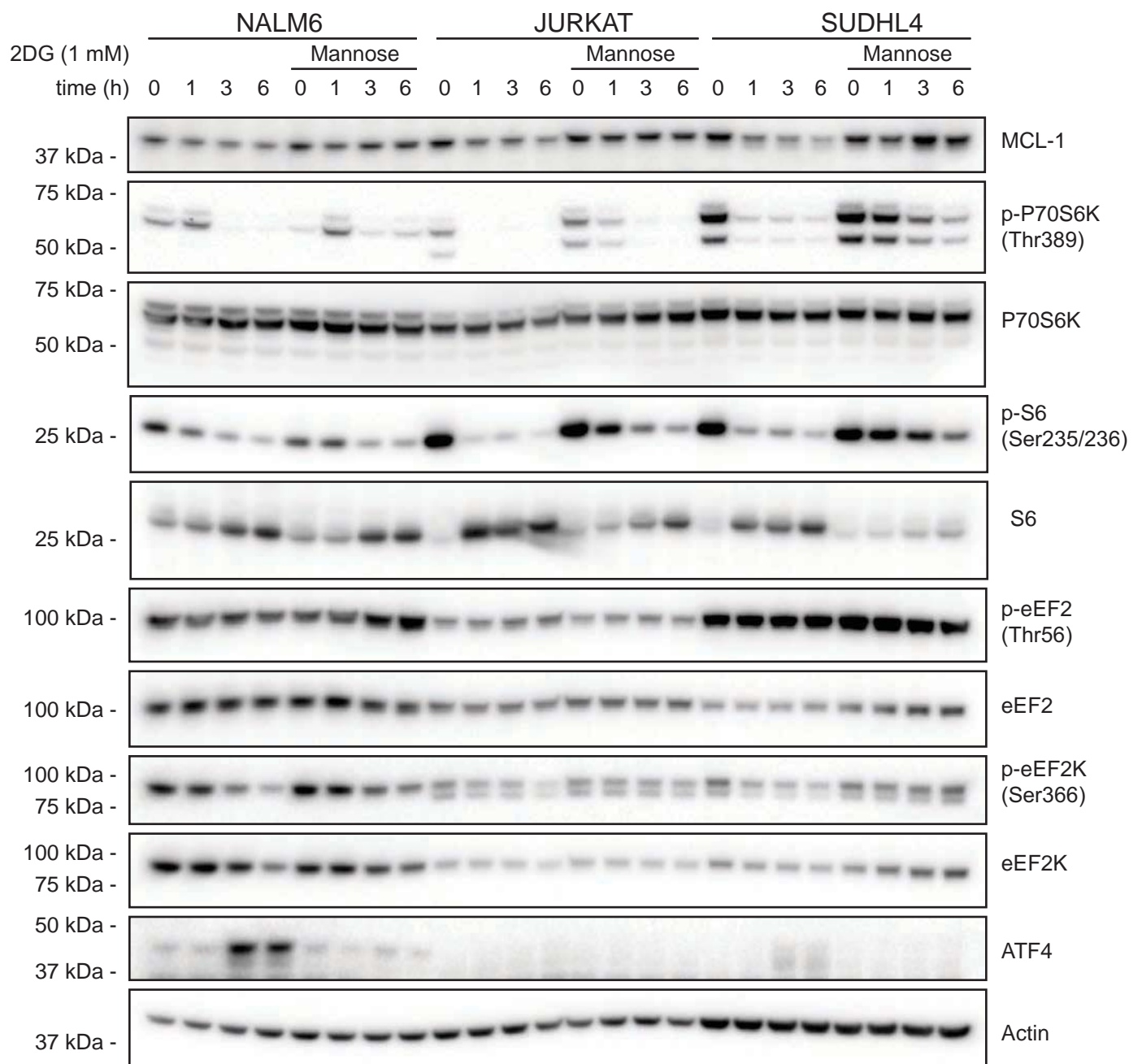

Supplement: Supplementary file 5 — Supplementary Figure 5 [file 41418_2018_244_MOESM5_ESM.pdf]

Supplementary Fig 6

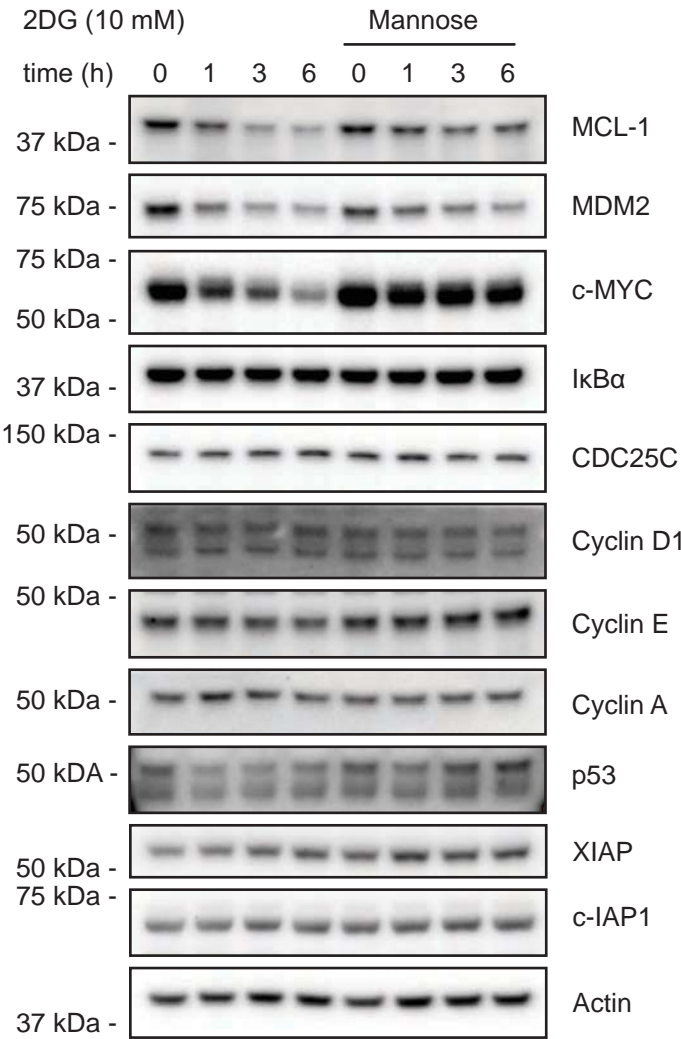

Supplement: Supplementary file 6 — Supplementary Figure 6 [file 41418_2018_244_MOESM6_ESM.pdf]
