# Supplementary material for: By reducing global mRNA translation in several ways, 2-deoxyglucose lowers MCL-1 protein and sensitizes hemopoietic tumor cells to BH3 mimetic ABT737
Source: Cell Death Differ. 2018 Dec 11;26(9):1766–81. doi: 10.1038/s41418-018-0244-y (PMC6748140; doi:10.1038/s41418-018-0244-y)
Supplement: Supplementary file 7 — supplementary figure legends [file 41418_2018_244_MOESM7_ESM.docx]

**Supplementary Figure Legends**

**Supplementary Figure 1. Co-treatment or pre-treatment with 2DG enhances the extent of caspase activation induced by ABT737**

(**A**) Immunoblots showing that cooperativity of 2DG and ABT737 in caspase-driven apoptosis develops within 2-3 hours. Treating NALM-6 cells with 1 mM 2DG alone for 6 h did not activate caspase-3 or capsase-8, nor BID cleavage. ABT737 (0.5 μM) alone induced limited activation of caspace-3 and limited BID cleavage, followed at 5-6 h by caspase-8 activation (its cleavage). Co-treatment with 2DG and ABT737 gave more extensive activation of caspases 3 and 8 and cleaved PARP, as did pre-treatment with 2DG for 3 h followed by the indicated period of ABT737 treatment. Notably, since active BID (tBID) has appeared only when caspase-3 is activated, most likely the tBID is a consequence rather than cause of caspase activation.

(**B**) FACS analysis of cell death induced in NALM-6, JURKAT and SUDHL-4 cells after 24 h by 0 to 20 mM 2DG, in presence or absence of mannose (10 mM). Data are plotted as mean ±SD. Significance determined by Two-Way ANOVA: *P<0.05, **P<0.01, ***P<0.001, ****P<0.0001.

(**C**) Cytochrome c release from mitochondria revealed by immunoblotting of NALM-6 cells treated with 2DG (1 mM), ABT737 (0.5 μM) or both, and Q-VD (50 μM) or mannose (10 mM) as indicated. After 3 or 6 h of treatment, a cytoplasmic fraction was resolved by centrifugation into cytosol (Supernatant) and mitochondrial component (Pellet).

(**D**) PARP cleavage, a classic apoptosis hallmark, was evaluated by western blotting of NALM-6 cells treated 6 h with 2DG (1 mM), ABT737 (0.5 μM) or both, and mannose (10 mM) or Q-VD (50 μM) as indicated. Actin served as a loading control.

**Supplementary Figure 2. Kinetics of the 2DG effects.**

(**A**) Western blot analysis of the kinetics of the stability of the pro-survival proteins MCL-1, BCL-2 and BCL-XL treated with 2DG (1 mM) at 1, 3 and 6 h, and the effect of mannose (10 mM) co-treatment, showing that the drop in MCL-1 is evident by 3 h.

**(B)** Western blot analysis showing that 2DG treatment for 1, 3 and 6 h has little if any effect on the level of BCL-2 pro-survival relative BCL-W.

(**C**) The impact of proteasome inhibition on the level of MCL-1 protein in NALM-6 cells treated with 2DG. After 6 h of 2DG treatment (1 mM or 10 mM), in the presence of MG132 (0.5 μM) or its absence, MCL-1 abundance was evaluated by western blotting. A representative western blot is shown, and the histogram below represents a quantification of 3 independent western blots, each normalized with actin. Data are plotted as mean ±SD. Significance determined by One-Way ANOVA: *P<0.05, **P<0.01, ***P<0.001, ****P<0.0001.

**(D)** FACS analysis of the cell cycle distribution of NALM-6 cells treated by 2DG (1 mM) for 1h, 3h or 6h, with or without mannose (10 mM). Data are plotted as mean ±SD of three independent experiments. One-Way ANOVA did not show any significant changes.

**Supplementary Figure 3. Effects of 2DG on the mTOR related pathway**

NALM-6 protein extracts were analyzed by western blotting at the indicated times of 2DG treatment, with or without mannose co-treatment. Key components of the mTOR pathway (mTOR, Raptor, PRAS, GSK3β, p70S6K, S6) and MAPK pathway (ERK, PDK1) are evaluated. The only reproducible changes observed have been in phosphorylation of S6, p70S6K, and eEF2K (see text).

**Supplementary Figure 4. Effects of the mTORC inhibitors torin-1 and rapamycin on the mTOR related pathway.**

Both drugs ablated phosphorylation of P70S6K and hence of S6, showing as expected that the mTORC1 complex is their principal positive regulator.

**Supplementary Figure 5. Western blotting of translational regulators showing that 2DG inhibits global protein synthesis similarly in NALM-6, JURKAT and SUDHL-4 cells.**

**Supplementary Figure 6. 2DG treatment markedly lowers the level of certain short-lived proteins but not others.**

An extended panel of short half-life proteins treated with 10 mM 2DG showing results similar to Figure 5F for MCL-1, MDMD2 and c-MYC.
